# Application of ultrasound-guided single femoral triangle and adductor canal block in arthroscopic knee surgery: a prospective, double-blind, randomized clinical study

**DOI:** 10.1186/s12871-024-02555-0

**Published:** 2024-05-23

**Authors:** Baizhou Chen, Minghe Tan, Qingshu Li, Siqi Wang, Daiyu Chen, Maoji Zhao, Jun Cao

**Affiliations:** The First Affiliated Hospital of Chongqing Medical University, 1 Youyi Road, Yuanjiagang, Yuzhong District, Chongqing, 400016 China

**Keywords:** Knee, Arthroscopic surgery, Analgesia, Nerve block, Humans, Pain, Postoperative pain

## Abstract

**Purpose:**

To compare the difference in analgesic effect between femoral triangle block (FTB) and adductor canal block (ACB) during arthroscopic knee surgery.

**Methods:**

Patients who underwent arthroscopic knee surgery were randomized preoperatively to FTB group or ACB group. For each group, 20 mL of 0.1% ropivacaine was injected. Primary outcomes: The numeric rating score (NRS) at 12 h after surgery at rest and during movement. Secondary outcome: (1) The NRS at post anesthesia care unit (PACU) and 2, 24 h after surgery at rest and during movement; (2) The quadriceps muscle strength at PACU and 2, 12, 24 h after surgery; (3) Consumption of Rescue analgesia; (4) Incidence of adverse reactions.

**Results:**

The NRS at 12 h after surgery at rest and during movement of ACB group were higher than FTB group. Among secondary outcomes, the NRS at PACU at rest and during movement, 2 h after surgery during movement of FTB group lower than ACB group; the quadriceps muscle strength at 2 h after surgery of FTB group stronger than ACB group. After multiple linear regression model analysis, the data showed additional statistically significant reduction NRS at 24 h after surgery at rest (0.757, *p* = 0.037) in FTB group. Other outcomes were similar between two groups.

**Conclusions:**

The FTB appears to provide superior pain control after knee arthroscopy than ACB, the FTB is superior to the ACB in quadriceps muscle strength at 2 h after surgery.

**Trial registration:**

The trial was registered in the Chinese Clinical Trial Registry (ChiCTR2300068765). Registration date: 28/02/2023.

## Introduction

Arthroscopic knee surgery is one of the most common orthopedic procedures. Adverse pain feedback after knee arthroscopy can seriously slow down the speed of early rehabilitation of patients, hinder the development of day surgery and significantly reduce patient satisfaction [1]. Arthroscopic knee surgery has been reported to cause moderate to severe pain [2], the presence of preoperative opioid use in such patients is a major risk factor for long-term opioid use after surgery. The opioid crisis has made research on optimizing analgesia in orthopedic surgery more important [3]. Regional block, as one of the main methods to reduce opioid abuse and accelerate the recovery of patients, has been paid more and more attention by clinicians [4–6].The ACB, as a motor nerve-sparing peripheral nerve block technique in knee joint surgery, mainly provides analgesia in the knee joint area by blocking the saphenous nerve (SN), and also has the benefits of protecting the quadriceps strength and shortening the length of hospital stay [7–9]. However, the location of the adductor canal (AC) is always controversial, The ACB mentioned in some studies is actually more appropriately called the FTB [9, 10].

There are anatomical differences between the two blocks. In the coronal plane, the proximal FT consists of the inguinal ligament, the lateral side consists of the medial side of the sartorius muscle, the medial side consists of the lateral side of the adductor longus muscle, and the junction of the sartorius and adductor longus was defined as the apex of the FT. The AC is a myofascial compartment in the middle and lower thigh that extends from the apex of the FT to the adductor hiatus [11]. The nerves distributed in the two areas are different. at the proximal end of the FT, one to three intermediate femoral cutaneous nerves branch from the femoral nerve. in the middle part of the FT is the nerve that feeds the vastus medialis muscle (VM), the medial femoral cutaneous nerve (MFCN) and the SN near the lateral aspect of the femoral artery [12]. In addition, nerve to the VM has its own fascial compartment, which separates from the saphenous nerve and the femoral artery. The SN is the only nerve consistently found inside the AC [13].

Therefore, we hypothesized that FTB would provide better analgesia effect and similar muscle strength protection than ACB.

## Materials and methods

The study was conducted in the First Affiliated Hospital of Chongqing Medical University from March 1, 2023 to May 31, 2023. This manuscript adheres to the Consolidated Standards of Reporting Trials (CONSORT) guidelines. This clinical trial research received approval of the Ethics Committee of the First Affiliated Hospital, Chongqing Medical University (Ethics Number: 2023-050). It was registered with the China Clinical Trial Registration Center on 28/02/2023 (Registration Number: ChiCTR2300068765), with the registration completed prior to the enrollment of any patients.

Inclusion criteria: (1) elective surgery for knee arthroscopy; (2) ASA grade I-III; (3) age 15–70 years; (4) patients under general anesthesia, signed informed consent and refused to use postoperative analgesia pump.

Exclusion criteria: (1) patients with NYHA ≥ III; COPD and lung function ≥ III grade; liver function Child-Pugh B and C grade; eGFR < 60 ml/min); (2) prolonged use of opioid analgesics or non-steroidal anti-inflammatory drugs for over one year; (3) patients with abnormal coagulation function; (4) patients with contraindications to local anesthetic drugs. (5) patients who cannot cooperate.

Eliminate criteria: (1) subjects withdrew informed consent without any reason; (2) loss of follow-up. (3) patients with postoperative neuropsychiatric disorders who could not cooperate.

All patients signed the informed consent before surgery with refused to use the postoperative analgesia pump. According to the SPSS25 software pre-generated random number table, this table contains the sequence of 1-100 and corresponding groups. Patients included in the trial were randomly divided into FTB group and ACB group according to the order of operation and corresponding random number table. Prior to grouping, the researcher informed the group operator of grouping assignments by using consecutively numbered, opaque, sealed envelopes. Group FTB or Group ACB had a preoperative ultrasound-guided single-injection with 20 ml of 0.2% ropivacaine.

### Blinding

aside from the regional anesthesiologist and the investigator, surgeons, theatre anesthesiologists, physiotherapists, nurses, caregivers, the data recorder, and data analysis were blinded to group allocation. Unmasking did not occur until statistical analysis was complete. Surgeons, operating room anesthesiologists, physical therapists, nurses, caregivers, cannot use the body surface after surgery to determine what type of block was performed. In our study, which was a single injection and no obvious covering to indicate where the procedure was performed.

### Preoperative FTB or ACB

**Fig. 1:**
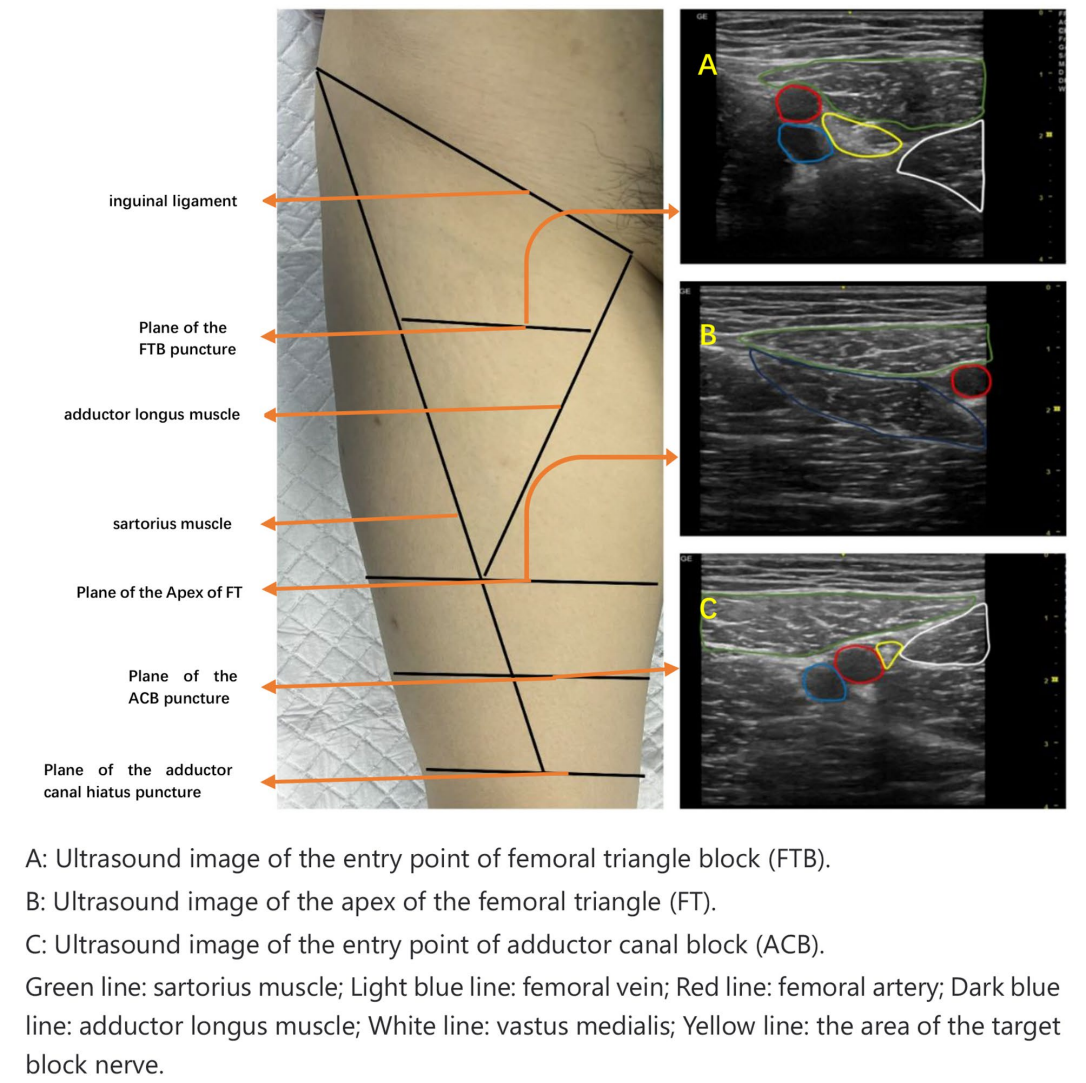
Ultrasound image of the plane of the puncture site

All patients entered the preparation room in advance, routine ECG monitoring, intravenous access and mask oxygen inhalation were established. During the nerve block, the patient was placed in the supine position, the knee joint was slightly abduction, the leg was slightly external rotation. high-frequency linear array probe 12 L-RS (4.2–13 MHZ, array element 192, GE Healthcare) was used to find the apex of the FT as the distal end of the FT, the inguinal ligament as the proximal end of the FT. A suitable puncture plane was found along the midpoint of the distal and proximal lines toward the medial thigh, which was used as the puncture point of the FTB. In the same way, the apex of the FT is found as the entrance of the AC, then the femoral artery is extended down to explore the inside of the AC. When the femoral artery passes through the adductor canal hiatus, it is the exit of the AC. The midpoint of the exit-entry lines was used as the puncture point for AC. Aseptic and in-plane techniques were used, a 0.7*80 mm (22G) enhanced developing needle (F type, LEAPMED, CHN) was used to inject. regardless of whether the saphenous nerve was visualized or not, 2–3 ml of normal saline was injected via the needle for hydro-dissection, and proper needle tip placement within the FT or AC was confirmed. The local anesthetic drugs were prevented from misrunning into the blood vessels by intermittent withdrawal. The injectate was seen peri-arterially spreading around the femoral artery. Nerve blocks in all patients were performed by the same experienced anesthesiologist. The loss of pinprick sensation over the knee joint area within 15 min after the injection was deemed a successful block. Figure 1.

### Intraoperative period

After the block, the patient was transferred to the operating room. Midazolam 0.03 mg/kg, sufentanil 0.3 µg/kg, propofol 1.5-2.0 mg/kg, vecuronium 0.1 mg/kg or rocuronium 1 mg/kg were intravenously injected for induction of general anesthesia. The depth of anesthesia was maintained by continuous pumping of remifentanil, propofol, and inhalation of sevoflurane, the fluctuation of circulation was maintained less than 30% of the basal level. After surgery, the patient was extubated through PACU and returned to the ward.

### Postoperative period

All patients received intravenous infusion of flurbiprofen axetil for postoperative analgesia (50 mg, q12h.ivdrip).

### Remedial analgesic solution

If the NRS at rest was ≥ 5, Acetaminophen tramadol (37.5 mg, st.po) or tramadol injection (100 mg, st.ivdrip) was used as rescue analgesia within 24 h after surgery. If the pain was not significantly relieved or continued to worsen, acetaminophen tramadol (37.5 mg, st.po) could be re-administered within 6 h until the NRS at rest was < 5.

### Outcome measures

#### The primary outcome

The primary outcome was the numeric rating score (NRS) at 12 h after surgery at rest and during movement. The NRS is an 11-point scale ranging from 0 to 10. 0 is no pain, 10 is the worst pain, the NRS at rest was defined as the pain score measured while the patient was resting in bed, and the NRS during movement was the pain score measured while the patient was performing the Manual Muscle Testing (MMT) for muscle strength.

*Secondary outcomes*: (1) The NRS at post anesthesia care unit (PACU) and 2, 24 h after surgery at rest and during movement; (2) The quadriceps muscle strength at PACU and 2, 12, 24 h after surgery; (3) Consumption of Rescue analgesia; (4) Incidence of adverse reactions. The quadriceps strength is assessed using MMT method as follows: Grade 0, no muscle contraction; Grade 1, slight contraction but unable to move the joint; Grade 2, the joint can move horizontally but cannot resist the gravity of the lower limbs; Grade 3, can resist lower limb gravity, but not resistance; Grade 4, can resist lower limb gravity and can resist certain resistance; Grade 5, able to exercise against large resistance. The NRS and quadriceps muscle strength at rest and during movement were recorded by the data recorder (Data recording time can fluctuate ± 30 min, outcomes measures from 0 to 6 am were not recorded, patients who discharged within 24 h after surgery obtain NRS and quadriceps muscle strength through WeChat video call). Rescue analgesic consumption during the first 24 h after surgery was transformed by the oral morphine equivalent (OME). The consumption of anesthetic drugs, vasoactive agents during the perioperative period, the consumption of rescue analgesics within 24 h after surgery and the incidence of adverse events were recorded.

### Sample size calculation

In our pilot study of 8 arthroscopic surgery patients with the ACB or FTB, the standard deviation of NRS was 1.65 after 12 h postoperatively. In our study the minimal clinically important difference (MCID) of the mean difference of NRS was 1.33 [14]. The sample size calculation for this study was based on two independent samples nonparametric tests with PASS15 software. According to the loss rate of follow-up of 20% in each group, 45 cases were needed to be included in each group.

### Statistical analysis

The investigator used SPSS25 software (IBM SPSS Statistics version 25.0, IBM©, Armonk, NY, USA) to analyze the data. The Shapiro-Wilk tests were used to confirm normality of the data distribution. Independent sample T test was used to analyze the data with normal distribution, Mann-Whitney U test was used to compare other data between the two groups. multiple linear regression models (Stata/MP17.0, Stata©, LLC4905 Lakeway Drive College Station, TX77845, USA) were used to examine the influence of independent variables such as gender, age, BMI, duration of surgery, and type of surgery on NRS. Continuous variables were presented as mean ± standard deviation (SD) or median with interquartile range (IQR). Categorical variables were expressed as numbers (percentages). Adjusted NRS were also presented as mean differences. two-sided tests with *p* < 0.05 were considered statistically significant.

## Results

**Fig. 2:**
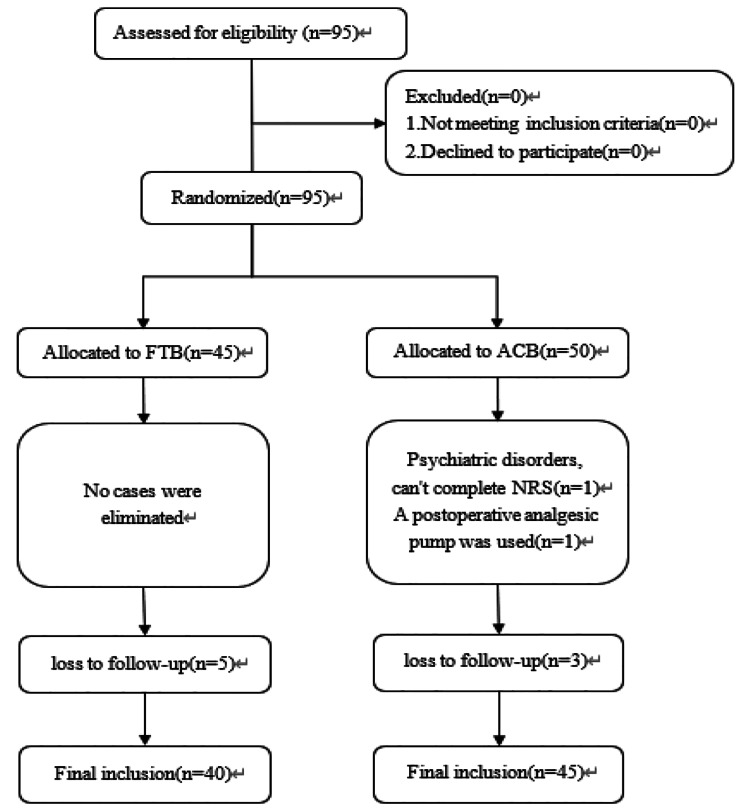
Flow diagram showing patient selection and randomization

95 patients who underwent knee arthroscopy from 1/3/2023 to 05/31/2023 were included. One patient was unable to complete the NRS after surgery due to long-term use of psychotropic drugs. Another patient was excluded who required the analgesic devices and informed consent was withdrawn. Three patients in ACB group and five patients in FTB group were lost to follow-up at 12 h after surgery for primary outcomes. At last, 85 effective cases were enrolled, including 40 cases in FTB group and 45 cases in ACB group Fig. 2. No statistically significant differences in baseline characteristics except BMI(FTB 23.06 ± 3.78 ***VS*** ACB 24.87 ± 4.04, *p* = 0.037) Table 1. The NRS at 12 h after surgery at rest and during movement of ACB group were higher than FTB group (NRS at 12 h after surgery during movement: FTB 2(1,3) ***VS*** ACB 3(2,5), *p* = 0.004; NRS at 12 h after surgery at rest: FTB1(0,2) ***VS*** ACB 2(0,4), *p* = 0.027). In secondary outcomes, the NRS at PACU at rest and during movement, 2 h after surgery during movement of FTB group lower than ACB group (NRS at PACU at rest: FTB 1(0,3) ***VS*** ACB 3(0,4), *p* = 0.047; NRS at PACU during movement: FTB 1(0,3.75) ***VS*** ACB 3(1,5), *p* = 0.016; NRS at 2 h after surgery during movement: FTB 2(1,3) ***VS*** ACB 3(1,5), *p* = 0.043); the quadriceps muscle strength at 2 h after surgery of FTB group stronger than ACB group (FTB 4(3,5) ***VS*** ACB 3.5(3,4), *p* = 0.042) Table 2. The adjusted NRS showed statistical differences at more moments (NRS at 24 h after surgery at rest, (0.757, *p* = 0.037)) Table 3. In the multiple linear regression model, there were no statistically significant differences in independent variables. There was no statistically significant differences in perioperative anesthetic and vasoactive drug consumption, rescue analgesic drug consumption, and adverse event rate between the two groups Table 4.

**Table 1:** Baseline characteristics

		FTB-group(*n* = 40)		ACB-group(*n* = 45)		*p*-value
Male, n (%)		14	35.00%	17	37.78%	0.794
ASA		2(2,2)		2(2,2)		0.289
Age at procedure, y		47.00(33.25,54.00)		49.00(34.50,59.00)		0.322
BMI, kg/m2		23.06 ± 3.78		24.87 ± 4.04		0.037
type of surgery, n(%)						0.912
	arthroscopic surgery	28	70.00%	32	71.11%	
	Invasive surgery	12	30.00%	13	28.89%	
time of operation, min		55.00(35.25,80)		60(42.50,90.00)		0.196

Data are shown as mean ± SD or median with interquartile range

Abbreviations: ACB, adductor canal block; FTB, femoral triangle block; SD, standard deviation; Arthroscopic surgery: knee arthroscopy, meniscal surgery; Invasive surgery: Cruciate ligament reconstruction, fracture surgery

**Table 2:** NRS score and quadriceps muscle strength after surgery

M(P25, P75), *N*	NRS at rest		*P*-value	NRS at movement		*P*-value	Quadriceps muscle strength		*P*-value
	FTB	ACB		FTB	ACB		FTB	ACB	
PACU	1(0,3), 40	3(0,4),44	0.047	1(0,3.75), 40	3(1,5),44	0.016	3.5(3,4), 40	3(3,4), 44	0.180
2 h after surgery	1(0,2), 33	2(0,4),32	0.118	2(1,3), 33	3(1,5),32	0.043	4(3,5), 33	3.5(3,4),32	0.042
12 h after surgery	1(0,2), 40	2(0,3),45	0.027	2(1,3), 40	3(2,5),45	0.004	4(4,4.75),40	4(3,4), 45	0.257
24 h after surgery	0.5(0,2),40	2(0,3),45	0.068	2.5(1,3.75),40	3(2,4),45	0.090	4(4,5), 40	4(4,4.5),48	0.556

Data are shown as median with interquartile range

Abbreviations: NRS, numerical pain rating scale; M, median; N, number of cases; ACB, adductor canal block; FTB, femoral triangle block

**Table 3:** Mean difference in NRS in adjusted multiple linear regression model

	NRS at rest	*P*-value	NRS at movement	*P*-value
PACU	0.867	0.037	1.057	0.023
2 h after surgery	0.887	0.066	0.986	0.048
12 h after surgery	0.860	0.023	1.187	0.005
24 h after surgery	0.757	0.041	0.557	0.119

Data are shown as mean differences

**Table 4:** Other data comparisons

M(P25, P75)	FTB(*n* = 40)	ACB(*n* = 45)	*P*-value ^b^
Preoperative NRS at rest	0(0,1.75)	0(0,0.50)	0.196
Preoperative NRS at movement	4(2,5)	3(2,5)	0.204
Preoperative QMS	5(4,5)	5(4,5)	0.458
Propofol consumption(mg)	217.86(187.62,252.49)	222.56 (179.72,304.43)	0.951
Remifentanil consumption(mg)	0.53(0.41,0.61)	0.53(0.41,0.88)	0.644
Vecuronium consumption(mg)	6.00(6.00,8.00)	8.00(6.00,8.50)	0.288
Sufentanil consumption(ug)	32.50(25.00,40.00)	30.00(25.00,40.00)	0.915
Noradrenaline consumption(mg)	0.10(0.00,0.16)	0.11(0.00,0.16)	0.871
Other vasoactive agents consumption	0.00(0.00,0.00)	0.00(0.00,0.00)	0.122
Oral morphine equivalent(mg)	0.00(0.00,0.00)	0.00(0.00,0.00)	0.502

Data are shown as median with interquartile range

Abbreviations: ACB, adductor canal block; FTB, femoral triangle block; SD, standard deviation; QMS, quadriceps muscle strength

## Discussion

In this study, the FTB group reported lower scores in the NRS during movement, and these differences were statistically significant at PACU, 2 h, 12 h after surgery; In terms of NRS at rest, the ACB group had a higher NRS than the FTB group at PACU, 12 h after surgery. However, at this moment of 24 h after surgery, the NRS of the two groups showed no statistical difference either at rest or during movement. After linear model adjustment, the FTB group showed superiority at 24 h at rest.

**Fig. 3:**
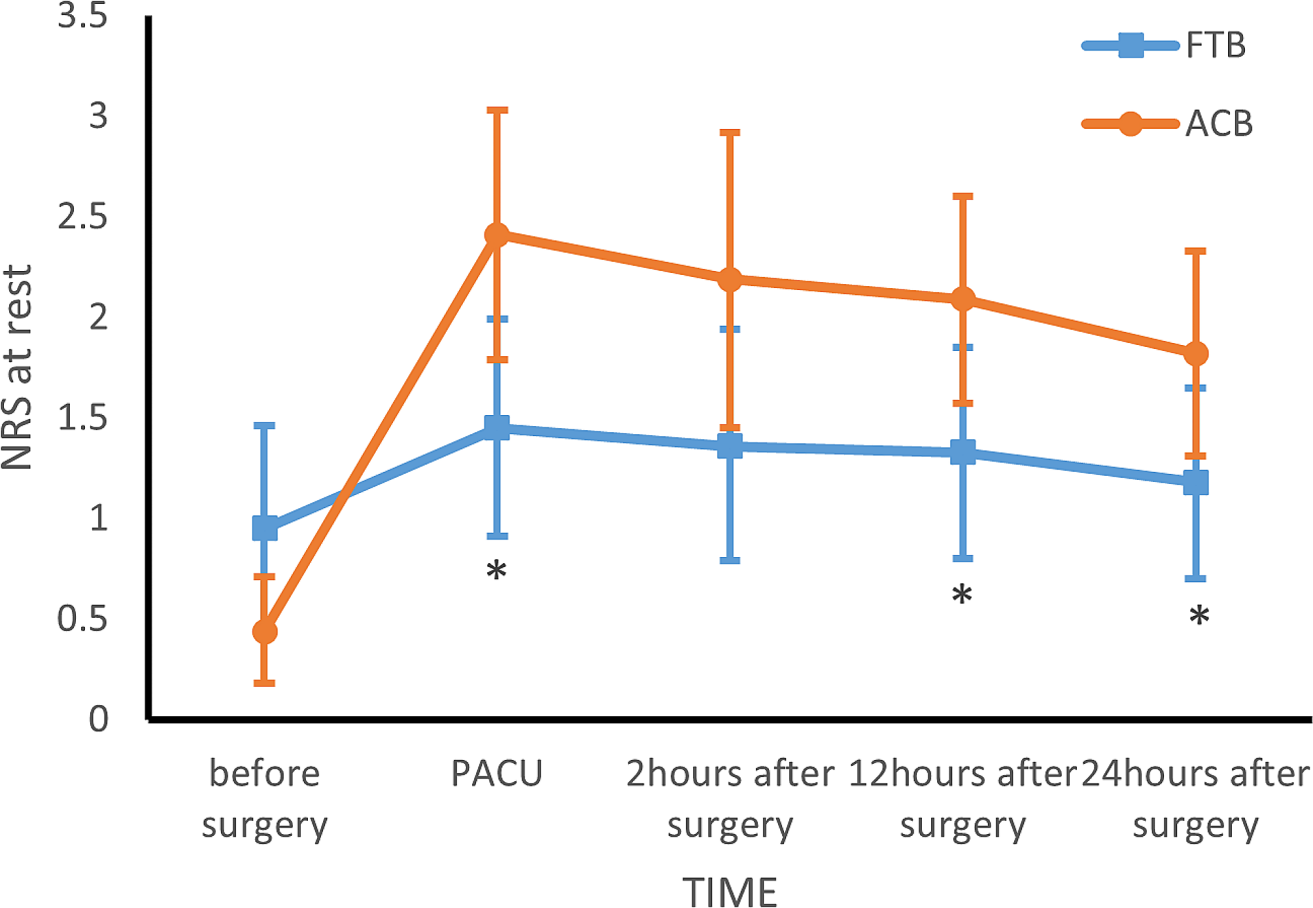
Mean NRS at rest with 95% CI per group over time. *p < 0.05

**Fig. 4:**
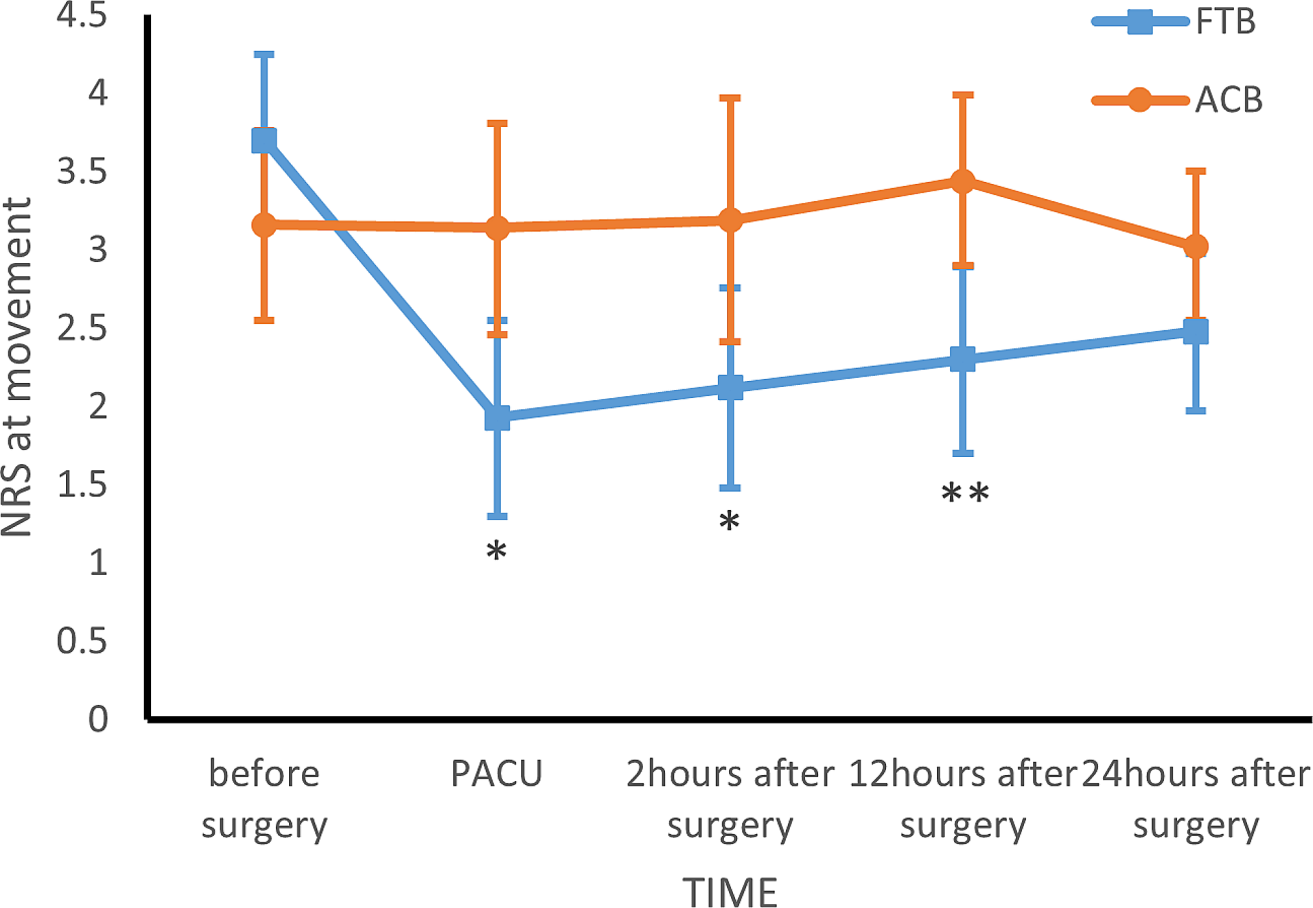
Mean NRS at movement with 95% CI per group over time *p < 0.05, **p < 0.01

The most important finding of this study was that the analgesic effect of the ACB and the FTB during arthroscopic knee surgery. Adjustment by the multiple linear regression model, we found that the mean of the NRS at any time points of the FTB group were lower than the ACB group when other variables were unchanged Figs. 3 and 4. However, in our study, the mean difference of NRS did not reach the MCIDTable 3, which means the analgesic effect of the two nerve block techniques with no obvious difference in clinical. Consistency with the results of the study was showed by Bora Lee et al [15].

As a branch of the femoral nerve (FN), the SN is a simple sensory nerve, as the main block nerve of the ACB, which is widely used in clinical practice. The nerve distribution of the adductor canal hiatus appears to be more complex. In this area, branches of the SN is involved in formation of patellar plexus [12]; Second, the nerve in the popliteal fossa distal, which is innervated the posterior and intra-articular structures of the knee joint [13, 16]. Some studies have pointed out that local anesthetic drugs can spread to the popliteal fossa during the ACB [17, 18], blocking nearby nerves and bringing analgesic benefits to patients. However, a case report suggested that the ACB may lead to impaired sciatic nerve function [19]. In other studies, the analgesic effect of the ACB were modest and clinically unimportant [2, 20, 21], Therefore, we believe that a single ACB may have limitations in the clinical setting.

Compared with the ACB, the FTB can affect more afferent branches of the FN [22], Like the SN, the vastus medialis nerve (VMN), the MFCN. The VMN and its terminal branches contain the sensory fibers of the knee joint [11, 12]. the muscular branch outlet of the VMN is now below the mid-thigh, this anatomical feature provides a theoretical basis for the FTB to minimize quadriceps weakness and maximize its analgesic effect [23]. In addition, after the MFCN originates from the proximal FT, it also gives off its anterior branch to innervate the skin on the medial side of the mid-upper thigh, its posterior branch follows the medial border of the sartorius muscle to innervate the skin on the medial side of the distal medial thigh. FTB has potential anatomical advantages.

The ideal volume of local anesthetic is to ensure that there is sufficient filling and that it does not spread to block the FN [13], which reduced quadriceps muscle strength. Sonawane K et al. mentioned the technique of high-volume proximal ACB, which reported immediate diffusion around the popliteal sciatic nerve and around the SN in the distal FT region after injection of local anesthetics, but not in the proximal FT or FN [24]. However, Giuseppe Pascarella et al. pointed out that there is an anatomical continuity between AC and FT, and that local anesthetics spread up and down AC or FT after injection [25]. In another study, Jæger P et al. also noted that there was no correlation between the volume of local anesthetic injected into the AC and the proximal spread of the FT. Anatomical structure may be a more important influencing factor. At the same time, for ACB, the dose of local anesthetic required to ensure distal extension of the adductor canal without a significant difference in quadriceps strength was 20 ml in their study [26].

However, the FTB is not perfect, the popliteal plexus and patellar plexus distal to the AC may not be anesthetized [16]. The popliteal fossa was also pointed out in a cadaveric study as an inaccessible area for the FTB [13]. This may be the reason why the FTB is less effective for pain management in the posterior part of the knee. Several studies have reported that block combined with obturator nerve or infiltration between popliteal artery and posterior genicular capsule results in better analgesia without affecting quadriceps muscle strength compared with single FTB [27, 28]. In addition, Hussain N et al. have shown that compared with other block techniques, regional anesthesia techniques targeting the distribution of femoral and sciatic nerves can provide the most consistent analgesic benefits [2]. This is also the direction of future research.

In several recent studies, the analgesic effects of the FTB and the ACB in total knee arthroplasty have been compared [9, 10, 29]. Similar to the results of this study, the FTB was associated with improved postoperative pain control and no negative effect on functional mobility compared with the ACB. Secondly, the location of the AC and the FT under ultrasound has been relatively clear. Compared with the ACB, the surface projection area of the FTB is wider, the FTB is easier and simpler for clinicians. In addition, relative to the FT, the AC is located lower and closer to the surgical area, which carries the risk of increased postoperative infection. Therefore, we believe that the FTB is a better block technique for arthroscopic knee surgery.

### Limitations

In this study, most procedures started after 20 o ‘clock or even close to 0 o ‘clock, resulting in a loss to follow-up rate of 10.5% within 12 hours after surgery for the primary outcome, it is also not possible to compare the differences in NRS and quadriceps muscle strength between the two block techniques during the period from 2 to 12hours after surgery through this study. Second, the quadriceps muscle strength was only measured by MMT in this study. It has been pointed out that the iliopsoas muscle plays a compensatory role in MMT, it would be more accurate to directly test the strength of the VM and vastus lateralis muscles [30]. in addition, the time of weight-bearing and active flexion of the knee joint were strictly prescribed by orthopedic surgeons for most patients, which was an important factor for the inability of most patients to complete the knee joint range of motion and results of the timed “up & go” tests. Therefore, our measurement of quadriceps strength may have been biased.

## Conclusions

In conclusion, compared with ACB, FTB can better control pain after knee arthroscopy in the early stage. In terms of muscle strength protection, FTB also showed some superiority in the early stage.

## Data Availability

The datasets used during the current study are available from the corresponding author on reasonable request.

## Abbreviations

AC: Adductor canal
ACB: Adductor canal block
FN: Femoral nerve
FNB: Femoral nerve block
FT: Femoral triangle
FTB: Femoral triangle block
IQR: Interquartile range
MCID: Minimal clinically important difference
MFCN: Medial femoral cutaneous nerve
NRS: Numeric rating score
OME: Oral morphine equivalent
ON: Obturator nerve
PACU: Post anesthesia care unit
SD: Standard deviation
MMT: Manual Muscle Testing
SN: Saphenous nerve
VM: Vastus medialis muscle
VMN: Vastus medialis nerve
